# Tween emulsifiers improved alginate-based dispersions and ionic crosslinked milli-sized capsules

**DOI:** 10.1038/s41538-023-00208-z

**Published:** 2023-06-27

**Authors:** Yulu Zheng, Ye Zi, Cuiping Shi, Huan Gong, Hongbin Zhang, Xichang Wang, Jian Zhong

**Affiliations:** 1 Xinhua Hospital, Shanghai Institute for Pediatric Research, Shanghai Key Laboratory of Pediatric Gastroenterology and Nutrition, Shanghai Jiao Tong University School of Medicine, Shanghai, China; 2 National R&D Branch Center for Freshwater Aquatic Products Processing Technology (Shanghai), Integrated Scientific Research Base on Comprehensive Utilization Technology for By-Products of Aquatic Product Processing, Ministry of Agriculture and Rural Affairs of the People’s Republic of China, Shanghai Engineering Research Center of Aquatic-Product Processing and Preservation, College of Food Science & Technology, Shanghai Ocean University, Shanghai, China; 3 Advanced Rheology Institute, Department of Polymer Science and Engineering, School of Chemistry and Chemical Engineering, Frontiers Science Center for Transformative Molecules, Shanghai Jiao Tong University, Shanghai, China

**Keywords:** Gels and hydrogels, Chemical engineering

## Abstract

The blending of surfactants might change the properties of alginate-based oil encapsulation preparations. Herein, the effects of Tween series (Tween 20, 40, 60, and 80) blending on the fish oil-encapsulated sodium alginate dispersions and calcium alginate capsules were studied. The results suggested Tween 80 showed better emulsifying properties than Span 80 for the alginate/surfactant emulsions. All the Tween series induced higher creaming stability than the sodium alginate-stabilized dispersion. Tween series blending did not change the sizes, decreased the water contents, and induced similar particle-like protrusions of calcium alginate capsules. Loading capacity and encapsulation efficiency of fish oil were dependent on the hydrophilic heads and fatty acid moieties of the Tween series. Tween series blending could increase the fish oil oxidative stability of the capsules. In the in vitro digestion process, Tween with saturated fatty acid moieties increased the free fatty acid release percentages. This work provided potential innovative processing technologies for improving the biological potency of fish oil.

## Introduction

Alginates are anionic natural polysaccharide salts of alginic acid from brown marine algae and two bacteria genera^1^. Due to their significant advantages (e.g., good renewability, biodegradation, antimicrobial activity, gelation capacity, and easy processing ability), alginates have been widely explored and used in the fields of tissue engineering, drug delivery, textile, cosmetics, and food science^2^. Especially, they have been widely explored as encapsulation materials to improve the stability or mask the unideal aroma of functional substances such as oil^3^, probiotics^4^, and flavor substances^5^.

Common oil encapsulation preparations include liquid dispersions^6^ and solid preparations (e.g., capsules)^7^. All liquid dispersions are thermodynamically unstable by nature^8^. The solid preparations are generally based on the ionic crosslinking of alginates with different cations such as Ca^2+^ ^9^. Calcium alginate capsules have been developed to encapsulate fish oil for protecting them against environments and isolating the fishy taste. Core–shell calcium alginate capsules were developed by a coaxial electrospraying technique to encapsulate fish oil/β-carotene^10^. External gelation-based multicore calcium alginate capsules were developed by a monoaxial electrospraying-external gelation technique to encapsulate fish oil^11,12^. In addition, internal gelation-based multicore calcium alginate capsules were developed by a monoaxial electrospraying-internal gelation technique to encapsulate fish oil^13^.

The multicore oil solid preparations generally included the preparation of oil emulsions/dispersions and then the gelation from the oil/water interface materials of the emulsions/dispersions to the wall materials of solid preparations. Oil-in-water emulsion is an important food system to disperse small oil droplets in water using emulsifiers^14,15^. Oil-in-water dispersion is a system to disperse small oil droplets in water using substances that are not emulsifiers^16^. The properties of the obtained oil encapsulation preparations are mainly dependent on the oil/water interface materials of liquid dispersions and wall materials of solid preparations. Therefore, the molecular blending of encapsulation materials by other chemicals such as polymers and small molecular surfactants might be an important strategy to improve the properties of alginate-based oil encapsulation preparations^17^.

Surfactants could improve the properties of alginate-based oil encapsulation preparations. Our recent work suggested that different Span surfactants (Span 20, 40, 60, and 80) had different effects on the fish oil-loaded alginate-based dispersions and capsules^18^. Tween series are typical small molecular surfactants for emulsion stabilization^19,20^. Though some food additives might induce gut microbiota dysfunction^21^, side effects of Tweens are occasionally reported in 1986 when Tweens were administrated intravenously with Vitamin E to low-birth weight infants^22^. Therefore, Tweens are generally recognized as safe and widely used as emulsifiers in food and pharmaceutics^23^. Tween 20 could increase the stability of dodecane-loaded alginate-based dispersions^24^. Tween 20 and 80 could increase the stability of sacha inchi oil-loaded alginate-based dispersions^25^. However, this work found that the emulsions with Tween 80 were not preferred for capsule preparation due to drop agglomerates^25^.

**Fig. 1:**
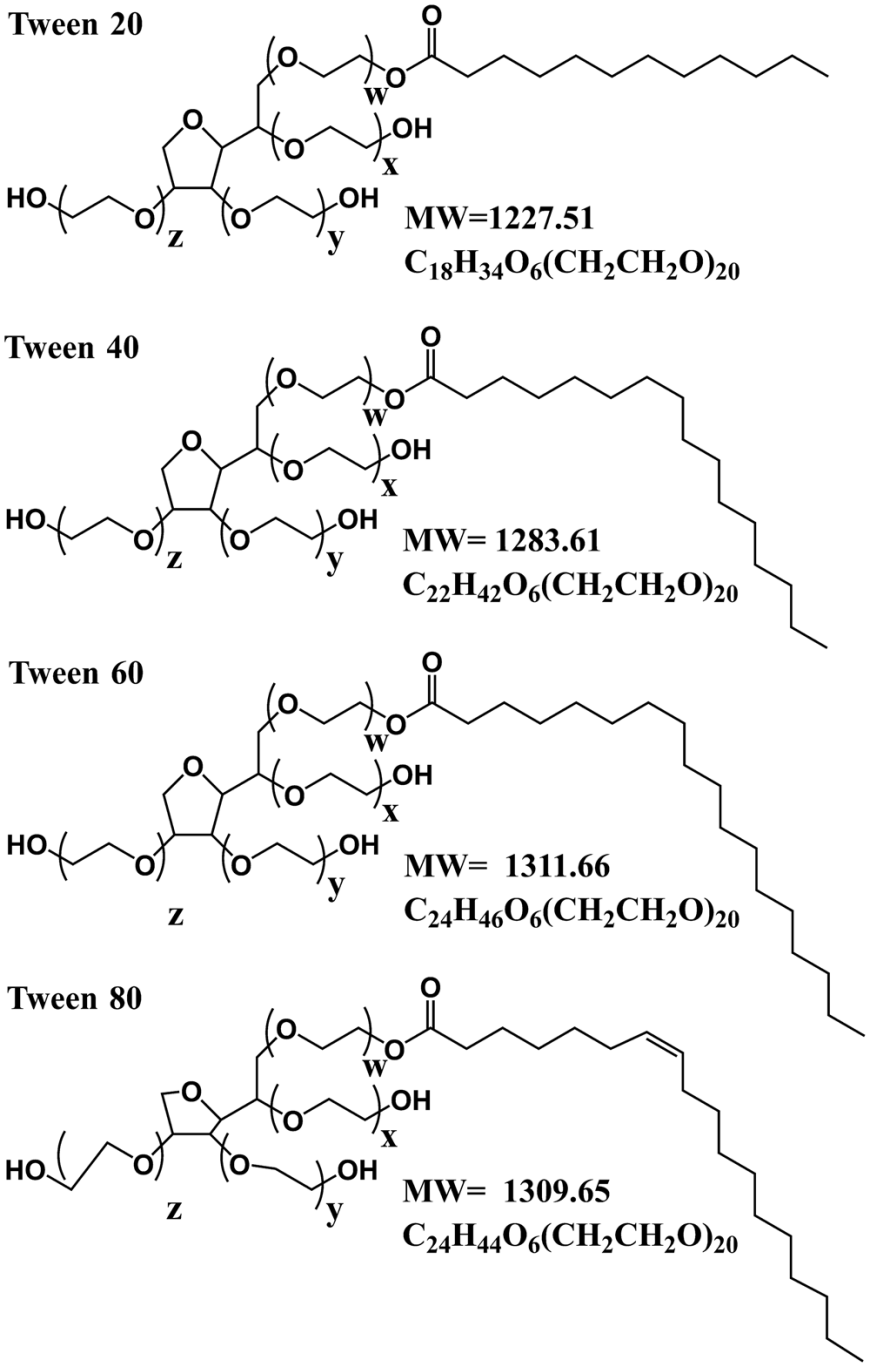
Molecular chemical structures of Tween series. The total number of polyoxyethylene subunits is 20 (x + y + z + w = 20).

Tween series are typical amphiphilic small molecules with similar hydrophilic heads and different hydrophobic fatty acid moieties, as shown in Fig. 1^26^. The hydrophilic and polar head consisted of a sorbitan ring connecting up to 4 polyoxyethylene (CH_2_CH_2_O) chains with variable lengths, and the fourth chain was esterified with hydrophobic fatty acid moieties^27^. The total number of polyoxyethylene subunits is 20 (x + y + z + w = 20). The fatty acid moieties for Tween 20, Tween 40, Tween 60, and Tween 80 are lauric acid, palmitic acid, stearic acid, and oleic acid, respectively. The molecular formulae of them are C_18_H_34_O_6_(CH_2_CH_2_O)_20_, C_22_H_42_O_6_(CH_2_CH_2_O)_20_, C_24_H_46_O_6_(CH_2_CH_2_O)_20_, and C_24_H_44_O_6_(CH_2_CH_2_O)_20_, respectively. Therefore, Tween series are good emulsifiers to analyze the effect of the chemical structure of the hydrophobic moieties structure on the properties of alginate-based oil encapsulation preparations.

## Results and discussion

### Analyses of sodium alginate/Tween 80-stabilized emulsions

**Fig. 2:**
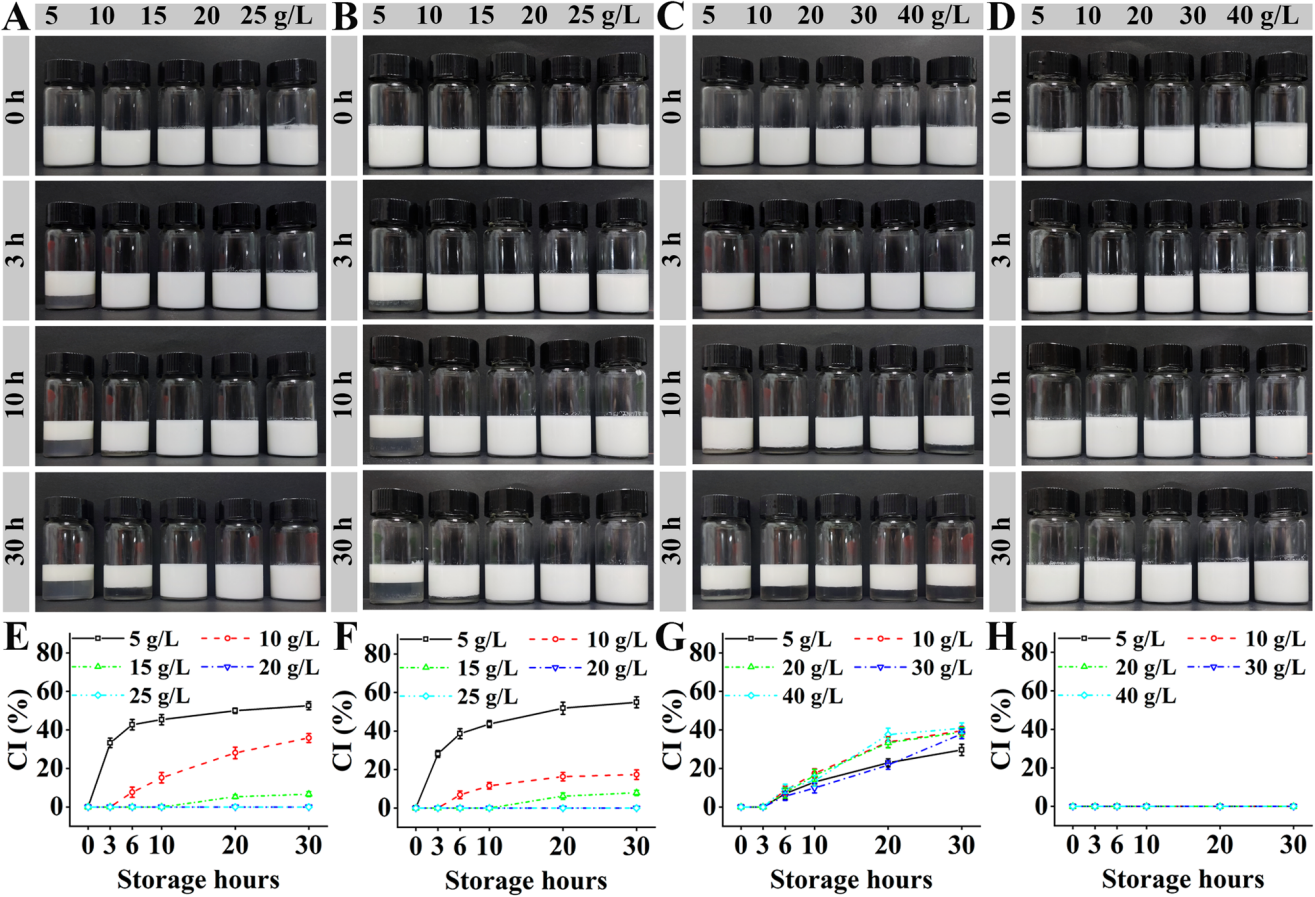
Stability of sodium alginate/Tween 80-stabilized emulsions at room temperature. **A** 10 g/L of Tween 80 and different concentrations of sodium alginate. **B** 20 g/L of Tween 80 and different concentrations of sodium alginate. **C** 10 g/L of sodium alginate concentration and different concentrations of Tween 80. **D** 20 g/L of sodium alginate concentration and different concentrations of Tween 80. **E**–**H** are the corresponding creaming index (CI) values to **A**–**D**.

Sodium alginate/Tween 80-stabilized emulsions were prepared with different concentrations and were observed during the storage at room temperature, as shown in Fig. 2. The freshly prepared emulsions were in milk white, which was similar to the fish oil-loaded alginate/Span-stabilized emulsions^18^. When the Tween 80 concentrations were 10 (Fig. 2A, E, and Supplementary Figure S1A) and 20 (Fig. 2B, F, and Supplementary Fig. S1B) g/L, the CI values increased with time and decreased with sodium alginate concentration. Moreover, ≥ 20 g/L of alginate induced zero CI values even after 30 h storage. When the concentration was 10 g/L (Fig. 2C, G, and Supplementary Fig. S1C), the CI values increased with time and Tween 80 concentrations showed no obvious effects on the CI values. When the concentration was 20 g/L (Fig. 2D, H, and Supplementary Fig. S1D), the CI values were zero even after 30 h and Tween 80 concentrations showed no obvious effects on the CI values. Therefore, sodium alginate concentration had higher effects on the emulsion stability than Tween 80 concentration.

The alginate/Tween 80 emulsions exhibited different behaviors (emulsion fluidity and creaming) to the alginate/Span 80 emulsions in our previous work^18^. It was interesting that no hollow holes with similar sizes to the 10-mm homogenizer head were present in the middle of all the emulsions (Images were not shown). The fluidity of alginate/Span 80 emulsions might decrease with the increasing concentrations of Span 80 and sodium alginate, and therefore the emulsions had hollow holes at relatively high emulsifier concentrations^18^. The interaction between Tween 80 and sodium alginate did not increase the fluidity of the emulsions even at high amounts of the emulsifier, which was different from Span 80. The sodium alginate/Tween 80-stabilized emulsions with 20 g/L of alginate and ≤ 10 g/L of Tween 80 (Fig. 2D) showed less CI values at 30 h than the alginate/Span 80 emulsion at the same concentrations in our previous work^18^. Therefore, Tween 80 might be the better emulsifier than Span 80 due to no obvious fluidity change and high creaming stability.

### Analyses of sodium alginate/Tween-stabilized emulsions

**Fig. 3:**
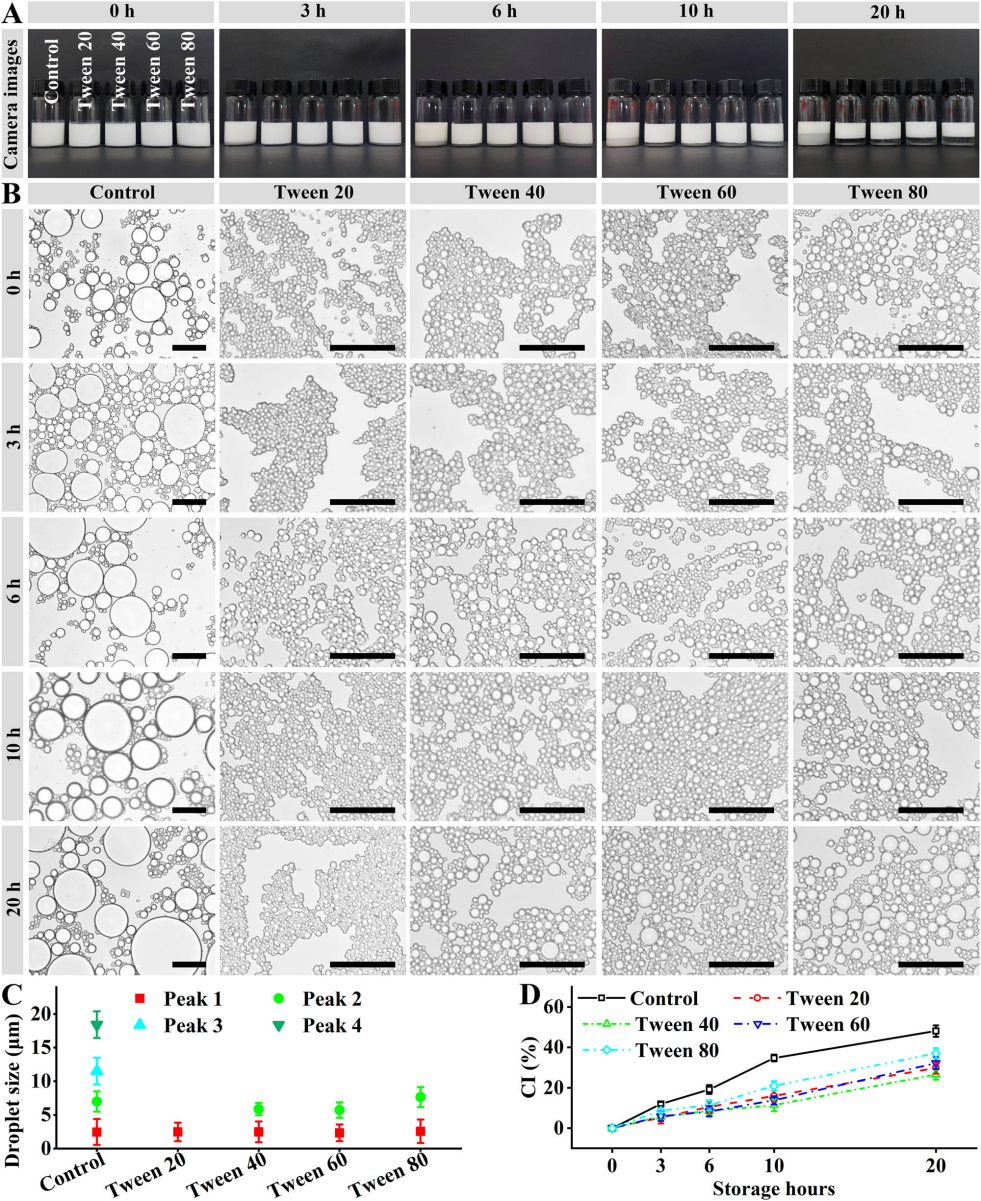
Stability of sodium alginate/Tween-stabilized emulsions. The concentrations of sodium alginate and Tween were 10 g/L. **A** Digital camera images. **B** Optical microscopy images. The black scale bars indicate 50 μm. **C** Most probable sizes in the freshly prepared emulsion droplets. **D** CI values of the emulsions.

Sodium alginate/Tween-stabilized emulsions were observed during the storage, as shown in Fig. 3. All the freshly prepared emulsions were in milk white and did not show obvious creaming. They were consistent with the sodium alginate/Tween 80-stabilized emulsions at different emulsifier concentrations (Fig. 2). All the emulsions consisted of microscale emulsions droplets (Fig. 3B). The sodium alginate dispersion showed quadrimodal droplet size distribution and some dispersion droplets with > 25 μm of sizes (Fig. 3C and Supplementary Figure S2A), which was similar to our previous publication^18^. The use of Tween significantly decreased the droplet sizes and modal distribution numbers (Fig. 3B, C, and Supplementary Figure S2B–E). Tween 20 induced monomodal droplet size distribution and others induced bimodal droplet size distribution.

With time, the droplets of the sodium alginate/Tween did not show obvious size changes (Fig. 3B), whereas showed significant creaming during the storage (Fig. 3A, D). All the sodium alginate/Tween-stabilized emulsions showed lower CI values than the sodium alginate-stabilized dispersion. For the dispersions or emulsions consisting of micron spherical droplets, CI is dependent on the move rate of the droplet (*V*_*stokes*_)^28,29^.

1 $${V}_{{stokes}}=-\frac{2\times {Gravity}\,{acceleration}\times {{Initial}\,{droplet}\,{radius}}^{2}\times \left({Droplet}\,{density}-{Water}\,{phase}\,{density}\right)}{9\times {\rm{Water}}\; {\rm{phase}}\; {\rm{viscosity}}}$$

2 $$\begin{array}{ll}\scriptstyle{Droplet\; density}\,\approx \,{\rm{Droplet}}\; {\rm{core}}\; {\rm{density}} \\ \qquad\qquad\qquad +\frac{3\,\times \,{\rm{Interfacial}}\; {\rm{layer}}\; {\rm{thickness}}\,\times \,({\rm{Interfacial}}\; {\rm{layer}}\; {\rm{density}}-{\rm{Droplet}}\; {\rm{core}}\; {\rm{density}})}{{Initial\; droplet\; radius}}\end{array}$$

The presence of Tween on the interfaces decreased the initial droplet sizes (Fig. 3B, C): sodium alginate/Tween 20 < sodium alginate/Tween 40 ≈ sodium alginate/Tween 60 < sodium alginate/Tween 80 < sodium alginate. However, due to the differences in fatty acid moieties (Fig. 1), the droplet core density and droplet density might be sodium alginate < sodium alginate/Tween 20 < sodium alginate/Tween 40 < sodium alginate/Tween 80 < sodium alginate/Tween 60. According to Equations (1 and 2), the CI values increased with the increase of the initial droplet radius (major factor) and the decrease of the droplet density (minor factor). Therefore, the final CI values were (Fig. 3D): sodium alginate/Tween 40 < sodium alginate/Tween 20 < sodium alginate/Tween 60 < sodium alginate/Tween 80 < sodium alginate. It should be noted that the sodium alginate/Tween-stabilized emulsions showed lower creaming stability than sodium alginate/Span 60- and sodium alginate/Span 80-stabilized emulsions^18^.

### Analyses of calcium alginate/Tween capsules

**Fig. 4:**
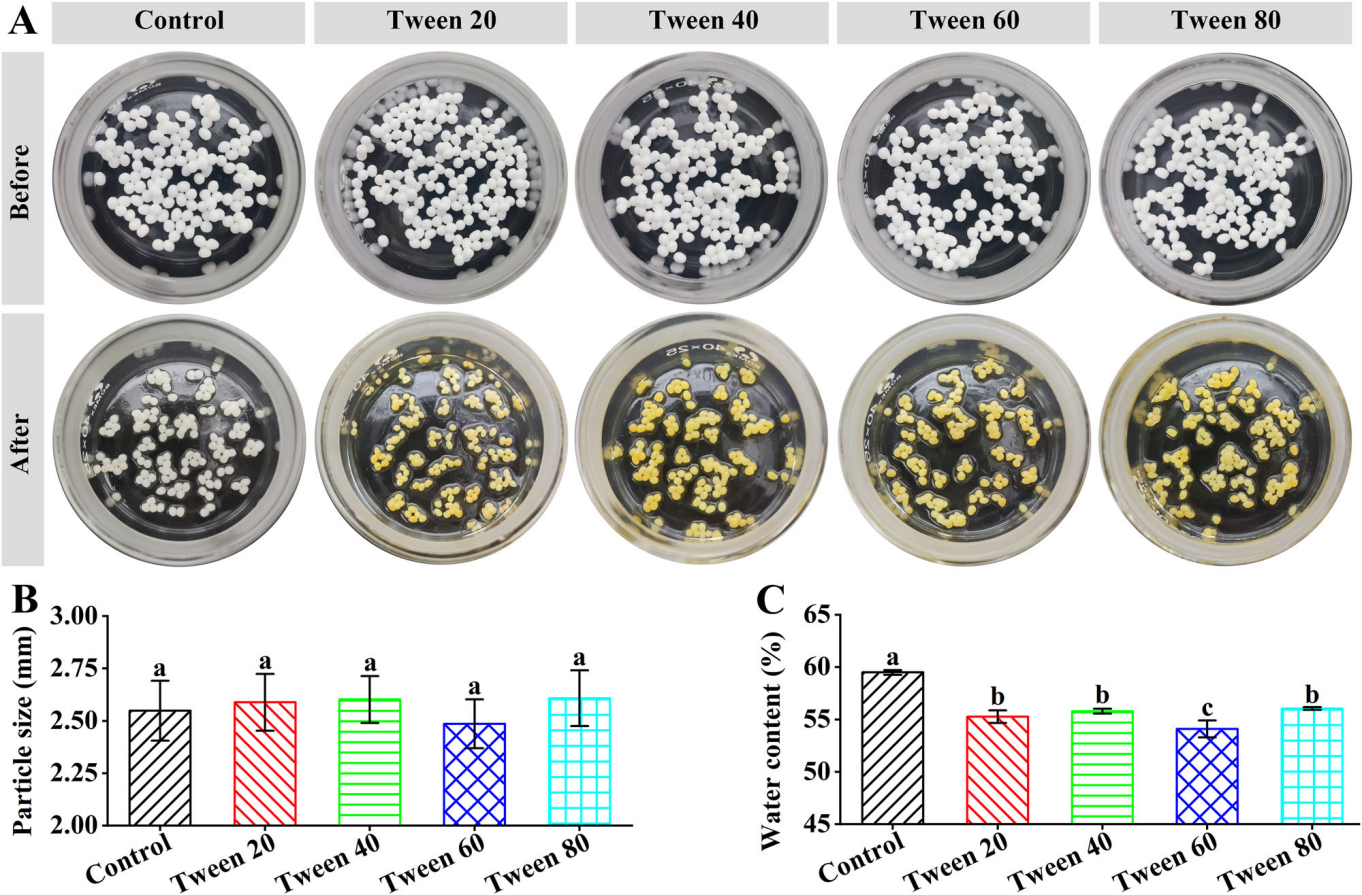
Capsule sizes and water content percentages of calcium alginate/Tween capsules. **A** Digital camera images before and after heating. The capsules were in glass vials with a height of 24 mm and a diameter of 40 mm. **B** Particle sizes. **C** Water content percentages. The error bar represents the standard deviation (*n* = 3). In each subFigure, significant differences (*p* < 0.05) were indicated by different letters.

By extruding sodium alginate/Tween-stabilized emulsions into CaCl_2_ solution, millimeter calcium alginate/Tween capsules could be prepared (Fig. 4A: Before). The capsules were white, which was consistent with sodium alginate/Span capsules^18^. The size of calcium alginate capsules was 2.55 ± 0.14 mm (Fig. 4B), which was similar to our previously prepared calcium alginate capsules^18^. The calcium alginate/Tween capsules showed similar sizes to the calcium alginate capsules and seems not to be dependent on the Tween series (Fig. 4B): Tween 20 (2.59 ± 0.14 mm) ≈ Tween 40 (2.60 ± 0.11 mm) ≈ Tween 60 (2.49 ± 0.12 mm) ≈ Tween 80 (2.61 ± 0.13 mm). Therefore, the presence of the Tween series in the capsules did not change the capsule sizes.

The water content percentages were determined by heating the capsules at 103 ± 1 °C for 2 h. After the heating process, the calcium alginate capsules became slightly yellow, whereas the calcium alginate/Tween capsules became bright yellow (Fig. 4A: After), which was different from the brown-yellow color of the electrosparyed capsules^11,13^. The capsules were not destroyed, which suggested that the Tween series could promote the diffusion of fish oil from the inner core to the surface of the capsules during the heating process. Further, as shown in Fig. 4C, the Tween series could decrease the water contents of the capsules (Significant difference < 0.05): calcium alginate capsules (59.5 ± 0.2%) > calcium alginate/Tween 80 capsules (56.1 ± 0.1%) ≈ calcium alginate/Tween 40 capsules (55.8 ± 0.2%) ≈ calcium alginate/Tween 20 capsules (55.3 ± 0.6%) > calcium alginate/Tween 80 capsules (54.1 ± 0.8%). It might result from the Tween blending-induced mass increase of the capsules.

**Fig. 5:**
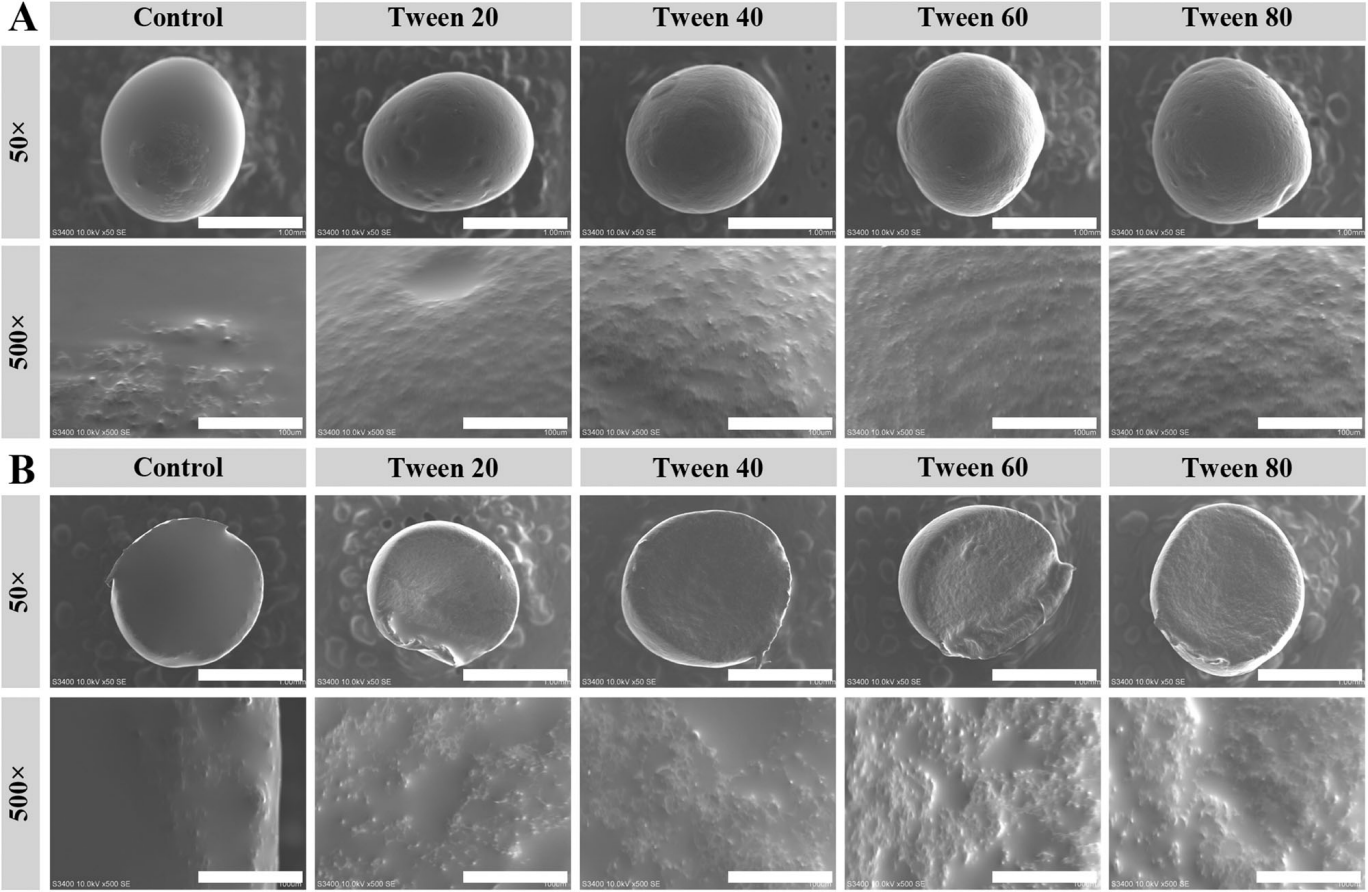
Scanning electron microscopy images with different magnifications of calcium alginate/Tween capsules. **A** Capsule surface. **B** Capsule section. The sizes of the white scale bars are 1 mm and 100 μm for the magnifications of ×50 and ×500, respectively.

According to the SEM images of the surface and section of the capsules, wrinkle-like protrusions appeared (Fig. 5: Control). It was consistent with our previous work^18^. The calcium alginate/Tween capsules showed similar particle-like protrusions. They were different from the calcium alginate/Span series capsules^18^. Therefore, the Tween series might be the main reason for the formation of the particle-like protrusions. Considering the Tween series had similar hydrophilic heads and different hydrophobic fatty acid moieties (Fig. 1)^26^, the hydrophilic heads might be the main reason for the formation of the particle-like protrusions for the calcium alginate/Tween capsules.

### LC, EE, and oil oxidative stability of calcium alginate/Tween capsules

**Fig. 6:**
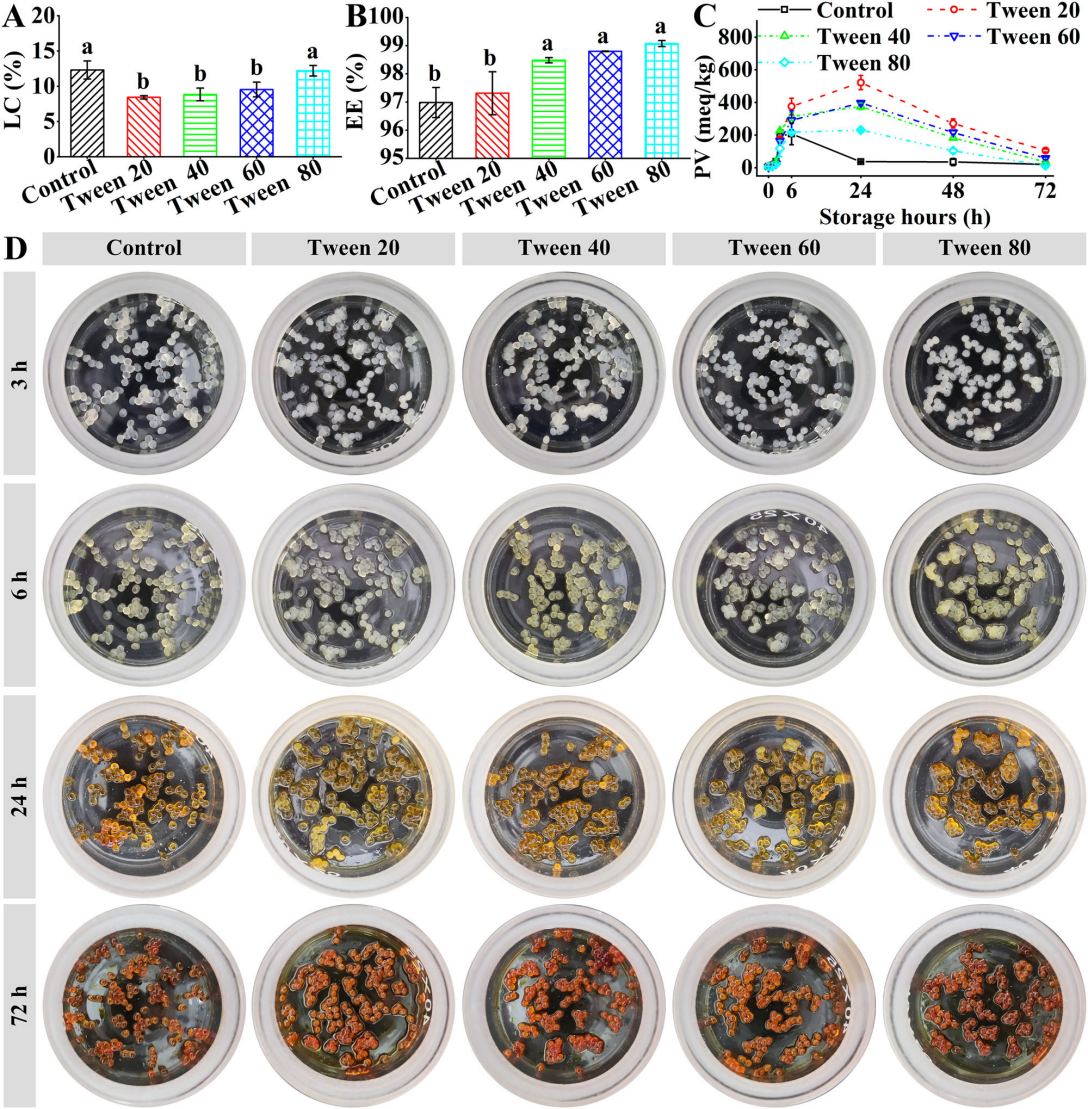
Fish oil characterization of calcium alginate/Tween capsules. **A** Loading capacity (LC). **B** Encapsulation efficiency (EE). In the subFigures, significant differences (*p* < 0.05) were indicated by different letters. **C** Peroxide value (PV) changes during the storage at 63 °C. **D** Digital camera images of the capsules during storage at 63 °C.

As shown in Fig. 6A, the LC values of the capsules were dependent on the Tween series (Significant difference < 0.05): Tween 20, Tween 40, and Tween 60 induced lower LC values than the calcium alginate capsules, whereas Tween 80 induced similar LC change to the calcium alginate capsules. The differences might result from the co-effect between Tween-induced mass increase and water content change-induced mass decrease (Fig. 4C) in the capsules. According to the average values, the LC values were dependent on both the hydrophilic heads and the fatty acid moieties of the Tween series: (i) The introduction of the Tween hydrophilic heads decreased the LC values; (ii) The LC values increased with the increase of molecular weights and double bond amounts of the fatty acid moieties. The LC values (8.4–12.2%) of the capsules were higher (Tween 80) than or similar (Tween 20, Tween 40, and Tween 60) to that (6–9%) of the fish oil-loaded electrospayed calcium alginate capsules^11,13^.

As shown in Fig. 6B, the EE values of the capsules were dependent on the Tween series (Significant difference < 0.05): Tween 40, Tween 60, and Tween 80 induced higher EE values than the calcium alginate capsules, whereas Tween 20 induced similar EE change to the calcium alginate capsules. According to the average values, the EE values were dependent on the fatty acid moieties of the Tween series: The EE values increased with the increase of molecular weights and double bond amounts of the fatty acid moieties.

The fish oil oxidative stability of the capsules was evaluated by determining the primary lipid hydroperoxides using a peroxide value (PV) measurement^30^, as shown in Fig. 6C, D. The capsule colors became yellow and then brown (Fig. 6D and Supplementary Figure S3). For the calcium alginate capsule, the PV values increased until 6 h and then decreased until 72 h. The trend was similar to that of the calcium alginate capsules^18^. The PV value after 6 h (207 meq/kg oil) was lower than that (308 meq/Kg oil) of the calcium alginate capsule after 3 h^18^ and similar to that (about 210 meq/Kg oil) of the calcium alginate capsules after 17 days at 37 °C^31^. The differences might result from different batch of fish oil. For the calcium alginate/Tween capsules, the PV values increased until 24 h, and then decreased until 72 h. Therefore, the secondary oxidation products were formed due to the conversion of the primary lipid hydroperoxides^32^. All the Tween series increased the PV values, and delayed the time of the highest peroxide value from 6 h to 24 h (Fig. 6C). It suggested that all the Tween series could inhibit the formation of the secondary oxidation products^33^, which was consisted with the Span series (Span 20, 40, 60, and 80)^18^. Therefore, all the Tween series and Span series could increase the fish oil oxidative stability of the capsules. The effect ability was dependent on surfactan type: Span 60 and Tween 80 showed the similar and the lowest PV values.

### In vitro digestion analyses

**Fig. 7:**
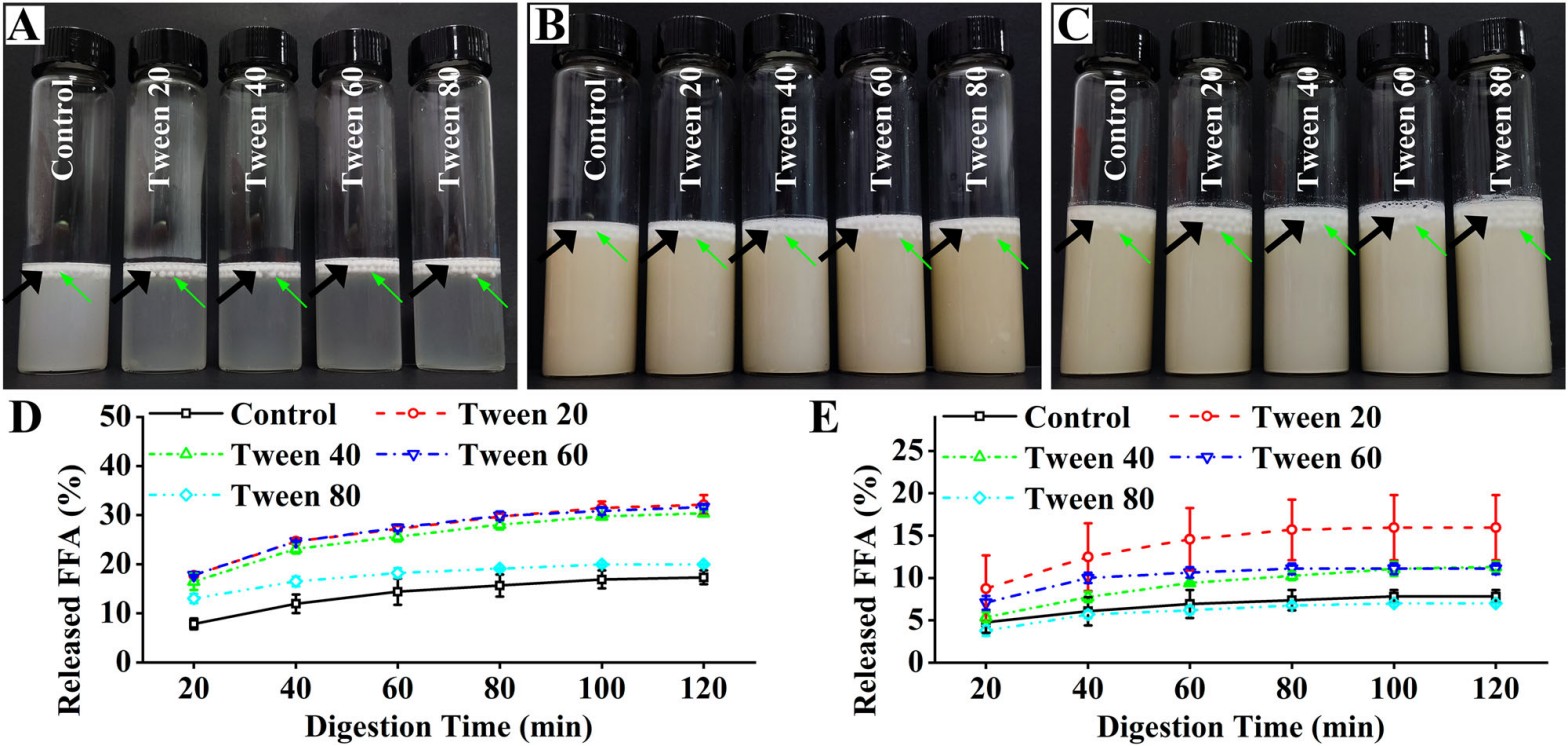
In vitro digestion behaviors of the capsules. **A**, **B** The capsules in the gastric phase at 2 h (**A**) and the small intestinal phase at 2 h (**B**) in the gastrointestinal tract model. **C** The capsules at 2 h in the small intestinal model. The capsule layers are indicated by black arrows. Single capsules are indicated by green arrows. **D**, **E** Free fatty acid (FFA) release behaviors in the small intestinal phase of the gastrointestinal tract model (**D**) and in the small intestinal model (**E**).

The in vitro digestion behaviors were studied in the gastrointestinal (Fig. 7A–B) and small intestinal (Fig. 7C) models. After the experimental process, the spherical structures of all the capsules were not destroyed, which was consistent with that of the calcium alginate/Span series capsules^18^. Therefore, the FFA release (Fig. 7D, E) was mainly attributed to the diffusion of fish oil from the surface of the capsules^34^, which also explained the final FFA release percentages were lower than 35% (Fig. 7D, E). It further confirmed the fish oil diffusion behaviors in the water content measurement.

During the gastrointestinal model (small intestinal phase), the FFA release percentages were dependent on the Tween series (Fig. 7D): Tween 20 ≈ Tween 40 ≈ Tween 60 > Tween 80 ≈ calcium alginate capsules. During the small intestinal model, the FFA release percentages were also dependent on the Tween series (Fig. 7E): Tween 20 > Tween 60 ≈ Tween 40 > Tween 80 ≈ calcium alginate capsules. Therefore, the saturated fatty acid moieties might increase the FFA release percentages of the capsules, whereas the unsaturated fatty acid moieties might have no obvious effect on the FFA release percentages.

In sum up, the effect of the Tween series on the oil-encapsulated sodium alginate dispersions and calcium alginate capsules was studied. All the results suggested the hydrophilic heads and fatty acid moieties had obvious effects on the structures and stability of sodium alginate/Tween-stabilized emulsions. Moreover, they also had obvious effects on the basic physicochemical properties, fish oil properties, and in vitro digestion behaviors of calcium alginate/Tween capsules. This work provided useful information to understand the effect of the Tween series on the preparation and properties of alginate-based oil encapsulation preparations. In the future, it is useful to explore the effect of the Tween series with modified heads or fatty acid moieties on the dispersions and capsules. In addition, it is also necessary to develop methods to allow the wall materials of the calcium alginate capsules to be disrupted to release 100% fish oil during the digestion process.

## Materials and methods

### Chemicals and reagents

Sodium alginate, Tween series, and porcine bile extract were purchased from Shanghai Macklin Biochemical Co. Ltd., China. Fish oil was purchased from Xi’an Qianyecao, Shaanxi Province, China. Lipase from porcine pancreas was purchased from Shanghai Lantuo Biotechnology Co., Ltd., Shanghai, China. Porcine gastric mucosa and other common reagents were purchased from Sinopharm Chemical Reagent Co., Ltd., Shanghai, China.

### Preparation of sodium alginate/Tween-stabilized emulsions

Sodium alginate and Tween were mixed in water (10 mL). Then, the sodium alginate/Tween solution and fish oil were mixed (3:1, v/v) and treated by a commercial T10 homogenizer (IKA, China)^35–37^. The homogenization speed was 11500 rpm. The homogenization time was 60 s.

### Emulsion characterization

The emulsions were photographed by a digital camera and an inverted microscope (MS600F, Shanghai Minz, China) at different times. The creaming index (CI) values were obtained as the height percentage of the serum layer to the total emulsion. The sizes of all the droplets from three randomly selected optical microscope images of different batches were measured. Gauss fitting multimodal distribution was performed to analyze the droplet sizes. The sodium alginate-stabilized dispersions were characterized as controls.

### Preparation of calcium alginate/Tween-stabilized capsules

The sodium alginate/Tween-stabilized emulsions were extruded into 100 mL of 15 g/L CaCl_2_ solution using a speed control pump^38^. The extrusion speed was 1 mL/min. A 20 Gauge of stainless needles was applied with an inner diameter of 0.60 mm. The CaCl_2_ solution was magnetically stirred at 300 rpm during the extrusion process. The collecting distance was 7 cm. The surrounding water of the obtained capsules was carefully removed before further characterization.

### Morphology observation of calcium alginate/Tween-stabilized capsules

A digital camera was applied to photograph the obtained capsules. Then, the sizes were measured using ImageJ 1.53c software (NIH, USA). The capsules were quickly cut after drying for 30 min. Then, the capsules were put on the conductive adhesive, sputtered, and observed using a scanning electron microscope (S-3400N, Hitachi, Tokyo, Japan)^37^.

### Water content percentage of calcium alginate/Tween-stabilized capsules

The capsules were heated in an oven (103 ± 1 °C) until the water content was completely evaporated. Then, the water content percentage was calculated as the percentage of the evaporated water content to the original sample mass.

### Loading capacity and encapsulation efficiency of the calcium alginate/Tween-stabilized capsules

The loading capacity (LC) of fish oil was determined according to the Rose-Gottlieb method^39^. The ammonia solution (1.25 mL, 25%) was mixed with the capsules (1.0–1.2 g, *m*_1_). The mixture was treated at 60 °C. After 5 min, the mixture was vibrated for 2 min. Then, ethanol (10 mL) was pipetted. Subsequently, diethyl ether (25 mL) and petroleum ether (25 mL) were added to the solution. After 30 min, the total volume (*V*_1_) of the ether layer in the liposuction bottle was measured. An ether part portion with a volume of *V*_2_ was added to a flask with a constant weight. The oil mass in the constant weight flask was determined by heating the sample at 102 ± 2 °C to a constant weight (*m*_2_)^18^. The LC (*L*_*C*_) of fish oil in the capsule was calculated according to the below equation:

3 $${L}_{C}( \% )=\frac{{\text{m}}_{2}{\text{V}}_{1}}{{m}_{1}{\text{V}}_{2}}\times 100$$

Encapsulation efficiency (EE) of fish oil in the calcium alginate/Tween-stabilized capsules was determined by measuring the surface oil mass and total oil mass^40^. The capsules (1.0–1.2 g, *m*_3_) were added in 10 mL of petroleum ether and vibrated for 2 min. Then, the mixture was filtered and the solution was collected in a constant-weight flask. This petroleum ether washing and filtering step was repeated twice and the solution was collected. Then, the filter paper was washed using 10 mL of petroleum ether. All the collected petroleum ether (40 mL) was the constant weight flask. The oil mass in the constant weight flask was determined by heating the sample at 102 ± 2 °C to a constant weight (*m*_4_)^18^. The EE was calculated according to the below equation:

4 $${EE}( \% )=\frac{{\text{m}}_{3}{\text{L}}_{c}-{\text{m}}_{4}}{{m}_{3}{\text{L}}_{c}}\times 100$$

### Oil oxidative stability evaluation

Using a Schaal oven condition, the oxidative stability of fish oil was evaluated^18,30^. Briefly, 0.5 g (*m*_5_) of the capsules were put in 20-mL glass vials and put in an oven (Temperature: 63 °C; RH: 70%) for 72 h. The morphologies of the treated capsules were photographed by a digital camera. Then, the peroxide values (*PVs*) were determined based on the Chinese National Standard “Determination of Peroxide Values in Food” (GB 5009.227-2016)^18^. The capsules were mixed with 30 mL of CH_3_COOH/(CH_3_)_2_CHCH_2_C(CH_3_)_3_ (volume ratio of 3:2). After 10 min, 1 mL of saturated KI (Shanghai Macklin) solution was pipetted to the mixture. After 3 min, ultrapure water (100 mL) was put in. Then, Na_2_S_2_O_3_ (0.001 mol/L, *c*) solution was carefully and slowly added until the yellow color vanished. Subsequently, 1 mL of 10 g/L starch indicator solution (Shanghai Macklin) was added. Na_2_S_2_O_3_ solution was added until the blue color vanished. The titrated volume (*V*_3_, mL) was recorded. The blank sample without the capsules was also titrated and the titrated volume (*V*_4_, mL) was recorded. PV was calculated according to below equation^31^:

5 $${PV}({meq}/{kg}\,{of}\,{oil})=1000\times \frac{({V}_{3}-{V}_{4})c}{{m}_{5}{L}_{C}}$$

### In vitro digestion behaviors of the capsules

The capsules (1.0 g) were studied in the gastrointestinal tract and small intestinal models^41^. If drinking with water, the time of the capsules in the mouth is generally a few seconds. Therefore, we did not study the capsule behaviors in the simulated mouth environment^13^.

For the gastrointestinal tract model, the capsules were treated in gastric and small intestinal phases. NaCl (2 g), HCl (37%, v/v%, 7 mL), and pepsin from porcine gastric mucosa (3.2 g) were mixed in water (1 L) and then pH was adjusted to 1.2. Then, 15 mL of the above solution was mixed with the capsules. The mixture was treated with NaOH to change pH to 2.0 and then vibrated at a speed of 100 rpm at 37 °C and a constant pH for 2 h (gastric phase). The mixture pH was changed to 7.0 and porcine bile extract solution (3.5 mL, 54 mg/mL) was pipetted. Then, 1.5 mL of CaCl_2_/ NaCl mixture (10 and 150 mmol/L, respectively) were added and the pH was changed to neutral. Subsequently, 2.5 mL of lipase from porcine pancreas in phosphate buffer was pipetted and the mixture was vibrated for 2 h (small intestinal phase).

For the small intestinal model, the capsules were treated in the small intestinal phase. The capsules were mixed with 15 mL water and the mixture pH was changed to neutral at 37 °C. Then, the samples were treated as same as the small intestinal phase in the gastrointestinal tract model experiments.

During the small intestinal phase, the pH was neutralized by 0.5 mol/L of NaOH (*V*_NaoH_) to neutralize the formed free fatty acid (FFA). The released FFA percentage can be calculated by assuming that one triglyceride molecule could release two FFA molecules:

6 $${\rm{FFA}}( \% )=\frac{{V}_{{\rm{NaoH}}}\times 0.5mol/L\times 868g/moL\,}{1.0g\times {L}_{C}\times 2}\times 100$$

### Statistical analysis

The data were expressed as average value ± standard deviation and were analyzed by One-way ANOVA followed by Duncan’s test (*p* < 0.05) in SPSS 27 software (International Business Machines Corp., Armonk, NY, USA).

### Reporting summary

Further information on research design is available in the Nature Research Reporting Summary linked to this article.

## Supplementary information

Supplementary materials

Reporting summary

## Data Availability

Data is available on request.

## References

[1] Ahmad Raus R, Wan Nawawi WMF, Nasaruddin RR (2021). Alginate and alginate composites for biomedical applications. Asian J. Pharm. Sci..

[2] Hurtado A, Aljabali AAA, Mishra V, Tambuwala MM, Serrano-Aroca Á (2022). Alginate: enhancement strategies for advanced applications. Int. J. Mol. Sci..

[3] Martins E, Poncelet D, Rodrigues RC, Renard D (2017). Oil encapsulation techniques using alginate as encapsulating agent: applications and drawbacks. J. Microencapsul..

[4] Li Z (2018). Biofilm-inspired encapsulation of probiotics for the treatment of complex infections. Adv. Mater..

[5] Lević S (2015). Characterization of sodium alginate/d-limonene emulsions and respective calcium alginate/d-limonene beads produced by electrostatic extrusion. Food Hydrocoll..

[6] Vandenbossche GMR, Remon J-P (1993). Influence of the sterilization process on alginate dispersions. J. Pharm. Pharmacol..

[7] Bennacef C, Desobry-Banon S, Probst L, Desobry S (2021). Advances on alginate use for spherification to encapsulate biomolecules. Food Hydrocoll..

[8] Reiner J, Walter EM, Karbstein HP (2023). Assessment of droplet self-shaping and crystallization during temperature fluctuations exceeding the melting temperature of the dispersed phase. Colloids Surf. A: Physicochem. Eng. Asp..

[9] Hu C, Lu W, Mata A, Nishinari K, Fang Y (2021). Ions-induced gelation of alginate: mechanisms and applications. Int. J. Biol. Macromol..

[10] Wang P (2020). Electrosprayed soft capsules of millimeter size for specifically delivering fish oil/nutrients to the stomach and intestines. ACS Appl. Mater. Interfaces.

[11] Tao L (2021). Shape control and stability of multicore millimetre-sized capsules using a combined monoaxial dispersion electrospraying–ionotropic gelation technique. Int. J. Food Sci. Technol..

[12] Tao L (2023). Preparation of multicore millimeter-sized spherical alginate capsules to specifically and sustainedly release fish oil. Food Sci. Hum. Wellness.

[13] Tao L (2022). Preparation and characterization of internal gelation-based electrosprayed multicore millimeter-sized fish oil-loaded calcium alginate-stabilized capsules. Food Hydrocoll..

[14] Zhang T (2021). Protein nanoparticles for Pickering emulsions: a comprehensive review on their shapes, preparation methods, and modification methods. Trends Food Sci. Technol..

[15] Zhang T (2020). Gelatins as emulsifiers for oil-in-water emulsions: extraction, chemical composition, molecular structure, and molecular modification. Trends Food Sci. Technol..

[16] Viegas IMA, Rinnan Å, Andersen SI (2023). Effect of isopropanol on the fluorescence of crude oil-in-water dispersions. Energy Fuels.

[17] Xiao S, Ahn DU (2023). Co-encapsulation of fish oil with essential oils and lutein/curcumin to increase the oxidative stability of fish oil powder. Food Chem..

[18] Zheng Y (2022). Effects of Span surfactants on the preparation and properties of fish oil-loaded sodium alginate-stabilized emulsions and calcium alginate-stabilized capsules. Int. J. Biol. Macromolecules.

[19] Zhang T (2020). Fish oil-loaded emulsions stabilized by synergetic or competitive adsorption of gelatin and surfactants on oil/water interfaces. Food Chem..

[20] Liu Z, Zhao M, Shehzad Q, Wang J, Sun B (2023). Whippable emulsions co-stabilized by protein particles and emulsifiers: the effect of emulsifier type. Food Hydrocoll..

[21] Luo M, Zhou D-D, Shang A, Gan R-Y, Li H-B (2021). Influences of food contaminants and additives on gut microbiota as well as protective effects of dietary bioactive compounds. Trends Food Sci. Technol..

[22] Kaur G, Mehta SK (2017). Developments of polysorbate (Tween) based microemulsions: preclinical drug delivery, toxicity and antimicrobial applications. Int. J. Pharmaceutics.

[23] Calicioglu M, Kaspar CW, Buege DR, Luchansky JB (2002). Effectiveness of spraying with tween 20 and lactic acid in decontaminating inoculated *Escherichia coli* O157:H7 and indigenous *Escherichia coli* Biotype I on Beef. J. Food Prot..

[24] Zhao Y, Chen Z, Wu T (2018). Cryogelation of alginate improved the freeze-thaw stability of oil-in-water emulsions. Carbohydr. Polym..

[25] Silva KFCE, da Silva Carvalho AG, Rabelo RS, Hubinger MD (2019). Sacha inchi oil encapsulation: Emulsion and alginate beads characterization. Food Bioprod. Process..

[26] Sannaningannavar FM, Patil SN, Melavanki RM, Navati BS, Ayachit NH (2014). Ultrasonic study of thermo-acoustic parameters of the polysorbate 20, 40, 60 and 80 liquid surfactants at different temperatures. J. Mol. Liq..

[27] Knoch H (2021). Complex micellization behavior of the polysorbates Tween 20 and Tween 80. Mol. Pharmaceutics.

[28] McClements DJ, Jafari SM (2018). Improving emulsion formation, stability and performance using mixed emulsifiers: A review. Adv. Colloid Interface Sci..

[29] Zhang T (2020). Octenyl succinic anhydride modification of bovine bone and fish skin gelatins and their application for fish oil-loaded emulsions. Food Hydrocoll..

[30] Chen J (2020). Comparative study on the evolution of polar compound composition of four common vegetable oils during different oxidation processes. LWT.

[31] Wu Q, Zhang T, Xue Y, Xue C, Wang Y (2017). Preparation of alginate core–shell beads with different M/G ratios to improve the stability of fish oil. LWT.

[32] Qiu X, Jacobsen C, Sørensen A-DM (2018). The effect of rosemary (*Rosmarinus officinalis L*.) extract on the oxidative stability of lipids in cow and soy milk enriched with fish oil. Food Chem..

[33] Nogueira MS, Scolaro B, Milne GL, Castro IA (2019). Oxidation products from omega-3 and omega-6 fatty acids during a simulated shelf life of edible oils. LWT.

[34] Wang P (2022). Electrospraying technique and its recent application advances for biological macromolecule encapsulation of food bioactive substances. Food Rev. Int..

[35] Liu G, Li W, Qin X, Zhong Q (2021). Flexible protein nanofibrils fabricated in aqueous ethanol: Physical characteristics and properties of forming emulsions of conjugated linolenic acid. Food Hydrocoll..

[36] Qin X (2022). Preparation of camellia oil pickering emulsion stabilized by glycated whey protein isolate and chitooligosaccharide: Effect on interfacial behavior and emulsion stability. LWT.

[37] Zhang T, Ding M, Wang X, Zhong J (2020). Droplet and creaming stability of fish oil-loaded gelatin/surfactant-stabilized emulsions depends on both the adsorption ways of emulsifiers and the adjusted pH. Food Sci. Hum. Wellness.

[38] Martins E, Renard D, Adiwijaya Z, Karaoglan E, Poncelet D (2017). Oil encapsulation in core–shell alginate capsules by inverse gelation. I: dripping methodology. J. Microencapsul..

[39] Bakry AM (2016). Stability of tuna oil and tuna oil/peppermint oil blend microencapsulated using whey protein isolate in combination with carboxymethyl cellulose or pullulan. Food Hydrocoll..

[40] Yang M (2023). Encapsulation of fish oil by complex coacervation and freeze drying with modified starch aid. Food Hydrocoll..

[41] Ding M (2021). Effect of interfacial layer number on the storage stability and in vitro digestion of fish oil-loaded multilayer emulsions consisting of gelatin particle and polysaccharides. Food Chem..

