# Supplementary material for: Tween emulsifiers improved alginate-based dispersions and ionic crosslinked milli-sized capsules
Source: NPJ Sci Food. 2023 Jun 27;7:33. doi: 10.1038/s41538-023-00208-z (PMC10300119; doi:10.1038/s41538-023-00208-z)
Supplement: Supplementary file 1 — Supplementary materials [file 41538_2023_208_MOESM1_ESM.docx]

**Tween emulsifiers improved** **alginate-based dispersions and ionic crosslinked milli-sized capsules**

Yulu Zheng^1,2^, Ye Zi^2^, Cuiping Shi^1^, Huan Gong^2^, Hongbin Zhang^3^, Xichang Wang^2^, Jian Zhong^1,2,^*

^1^Xinhua Hospital, Shanghai Institute for Pediatric Research, Shanghai Key Laboratory of Pediatric Gastroenterology and Nutrition, Shanghai Jiao Tong University School of Medicine, Shanghai 200092, China

^2^National R&D Branch Center for Freshwater Aquatic Products Processing Technology (Shanghai), Integrated Scientific Research Base on Comprehensive Utilization Technology for By-Products of Aquatic Product Processing, Ministry of Agriculture and Rural Affairs of the People's Republic of China, Shanghai Engineering Research Center of Aquatic-Product Processing and Preservation, College of Food Science & Technology, Shanghai Ocean University, Shanghai 201306, China

^3^Advanced Rheology Institute, Department of Polymer Science and Engineering, School of Chemistry and Chemical Engineering, Frontiers Science Center for Transformative Molecules, Shanghai Jiao Tong University, Shanghai, 200240, China

*Corresponding author: Xinhua Hospital, Shanghai Institute for Pediatric Research, Shanghai Key Laboratory of Pediatric Gastroenterology and Nutrition, Shanghai Jiao Tong University School of Medicine, Shanghai 200092, China

E-mail: jzhong@shsmu.edu.cn

Abbreviated running title: Tween emulsifiers improved alginate dispersions and capsules


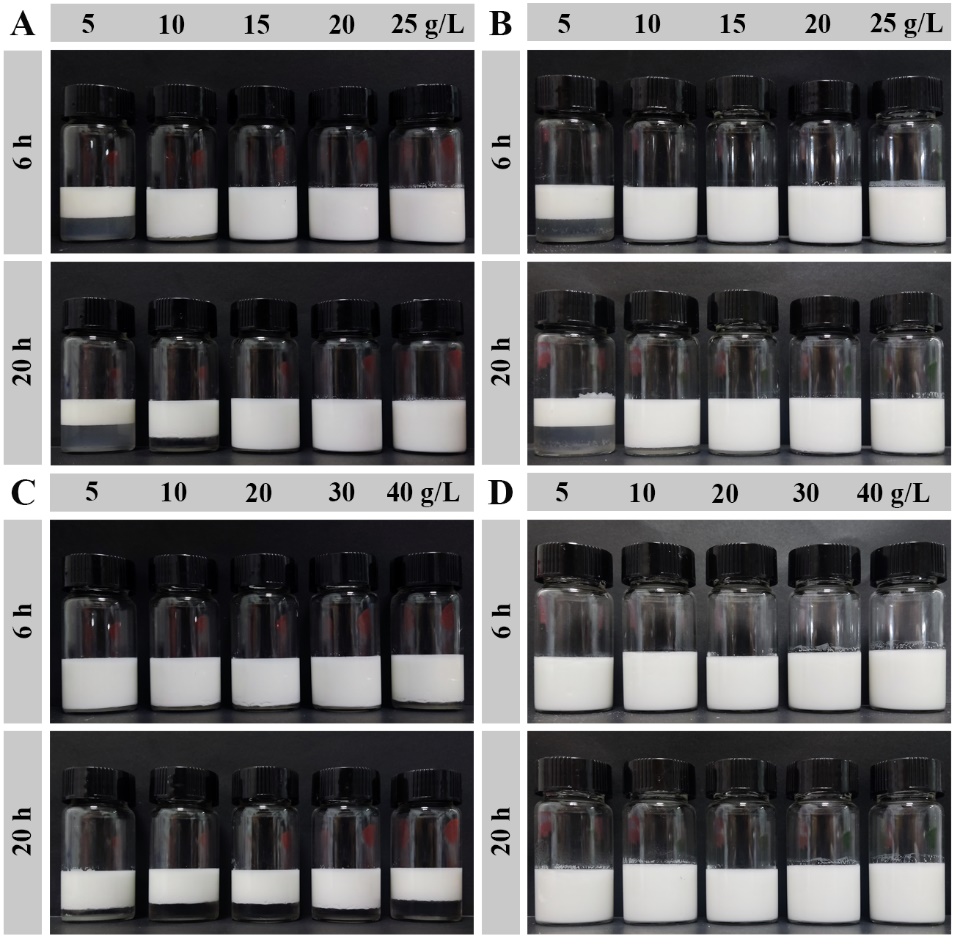


**Supplementary Figure 1.** Digital camera images of sodium alginate/Tween 80-stabilized emulsions at room temperature storage of 6 h and 20 h. (A): Tween 80 concentration of 10 g/L and different sodium alginate concentrations. (B): Tween 80 concentration of 20 g/L and different sodium alginate concentrations. (C): Sodium alginate concentration of 10 g/L and different Tween 80 concentrations. (D): Sodium alginate concentration of 20 g/L and different Tween 80 concentrations.

**
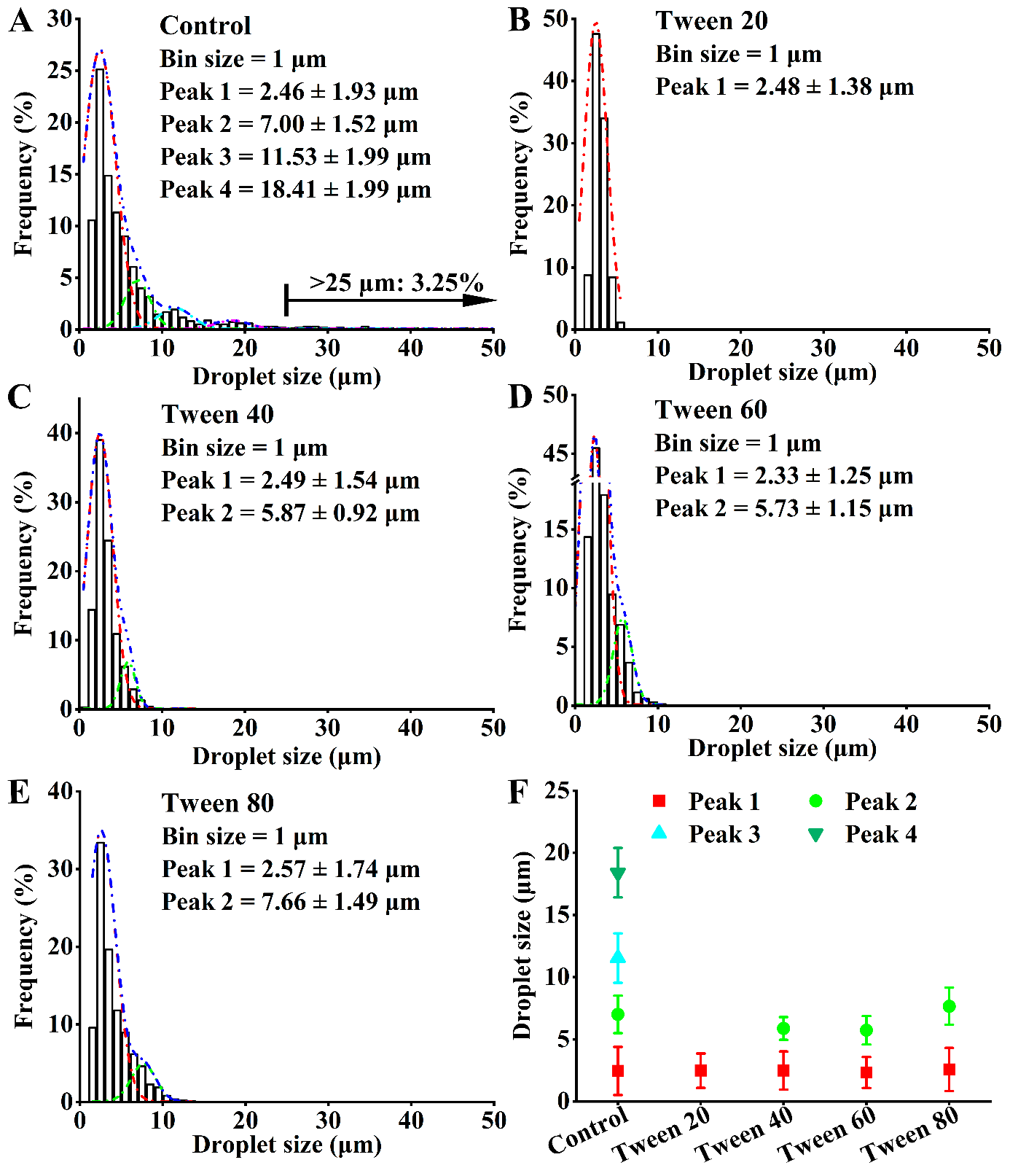
**

**Supplementary Figure 2.** Representative droplet size distribution in the freshly prepared sodium alginate/Tween series-stabilized emulsions (Fig. 3: 0 h). (A): Sodium alginate. (B): Sodium alginate/Tween 20. (C): Sodium alginate/Tween 40. (D): Sodium alginate/Tween 60. (E): Sodium alginate/Tween 80. (F): The most probable droplet sizes in the emulsions. Gaussian fit lines in (A–E) were applied to analyze the most probable droplet sizes.

**
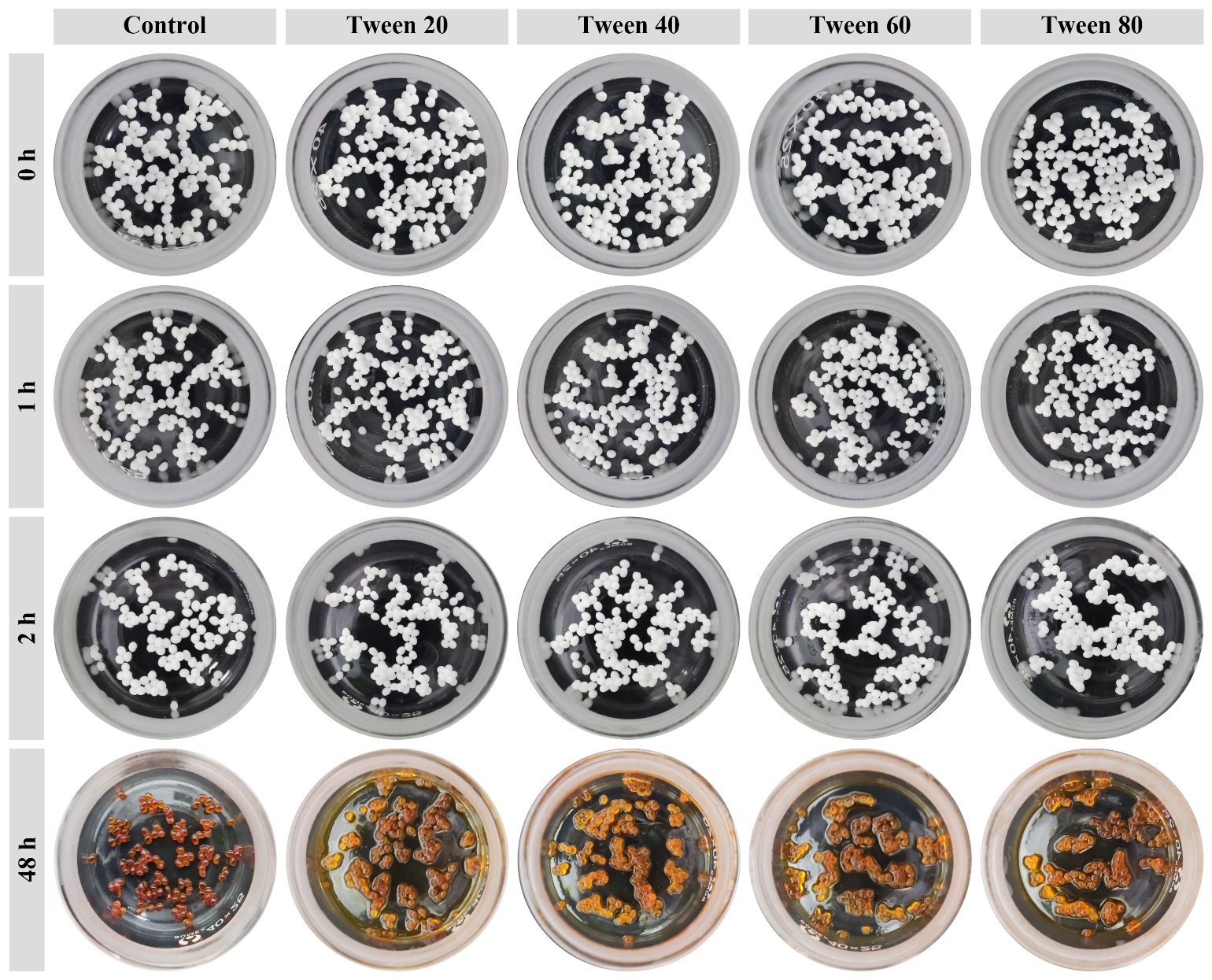
**

**Supplementary Figure 3.** Digital camera images of calcium alginate/Tween series capsules in glass vials at 63 ℃ with different incubation times.
